# Research on Detection of Ultra-Low Concentration Anthrax Protective Antigen Using Graphene Field-Effect Transistor Biosensor

**DOI:** 10.3390/s23135820

**Published:** 2023-06-22

**Authors:** Ting Liang, Jingfei Chen, Rui Yan, Huaning Jiang, Hexi Li

**Affiliations:** 1 The Institute of NBC Defense PLA Army, Beijing 102205, China; 2 Unit No. 32169 of PLA, Nyingchi 860000, China; dh18702314613@163.com; 3 Unit No. 32281 of PLA, Chengdu 610200, China; 4 Unit No. 31666 of PLA, Zhangye 610200, China

**Keywords:** graphene field-effect transistor, biosensor, anthrax protective antigen

## Abstract

Background: Protective antigen (PA) is an important biomarker for the early diagnosis of anthrax, and the accurate detection of protective antigen under extremely low concentration conditions has always been a hot topic in the biomedical field. To complete the diagnosis of anthrax in a timely manner, it is necessary to detect PA at extremely low concentrations, as the amount of PA produced in the early stage of anthrax invasion is relatively small. Graphene field-effect transistor (Gr−FET) biosensors are a new type of material for preparing biosensors, with the advantages of a short detection time and ultra-low detection limit. Methods: The effect of different concentrations of diluents on the affinity of PA monoclonal antibodies was determined via an ELISA experiment. Combined with the Debye equation, 0.01 × PBS solution was finally selected as the diluent for the experiment. Then, a PA monoclonal antibody was selected as the bio-recognition element to construct a Gr−FET device based on CVD-grown graphene, which was used to detect the concentration of PA while recording the response time, linear range, detection limit, and other parameters. Results: The experimental results showed that the biosensor could quickly detect PA, with a linear range of 10 fg/mL to 100 pg/mL and a detection limit of 10 fg/mL. In addition, the biosensor showed excellent specificity and repeatability. Conclusions: By constructing a Gr−FET device based on CVD-grown graphene and selecting a PA monoclonal antibody as the bio-recognition element, a highly sensitive, specific, and repeatable Gr−FET biosensor was successfully prepared for detecting extremely low concentrations of anthrax protective antigen (PA). This biosensor is expected to have a wide range of applications in clinical medicine and biological safety monitoring.

## 1. Introduction

Anthrax is a severe infectious disease caused by the Gram-positive rod-shaped bacterium Bacillus anthracis [1]. It is easily spread through infected animals or contaminated animal excreta, and over one fifth of the world’s population is at high risk of anthrax infection [2]. Due to its ability to survive for long periods in soil and its ease of spread in nature, Bacillus anthracis is often used as an effective biological weapon, posing a serious threat to global biosecurity [3]. If used in a terrorist attack, the consequences would be unimaginable.

The diagnosis of anthrax in the early stages of the disease is difficult due to the lack of typical clinical manifestations [4]. Laboratory testing is required to confirm the diagnosis, and the detection of anthrax protective antigen (PA) has been found to be an important biological marker for anthrax [5]. However, existing PA detection technologies include enzyme-linked immunosorbent assays (ELISAs) [6], immunomagnetic beads (IMBs) [7,8], homogenous time-resolved fluorescence (HTRF) [9,10], and biosensors [11,12,13,14], which can only detect nanogram- or picogram-level concentrations of PA and have problems such as cumbersome operation steps, a large instrument volume, and high consumable prices that cannot be solved in the short term.

In recent years, it has become a research focus to use new materials to prepare biosensors for detecting biomarkers at lower concentrations [15]. In 2021, Singh et al. proposed an unlabeled biosensor based on a charged plasma cylindrical gate nanowire tunneling field-effect transistor, which has the characteristics of low power consumption and high sensitivity [16]. With further research, graphene field-effect transistor (Gr−FET) biosensors have been widely studied due to their unique advantages, such as their short detection time, ultra-low detection limit, small size, and strong integrated circuit compatibility [17], becoming one of the important directions for the future development of biosensors [18].

Researchers have selected appropriate crosslinking agents in tumor biomarker detection, and fixed bio-recognition elements (monoclonal antibodies, single-chain antibodies, etc.) to the graphene surface through covalent or various non-covalent interactions, using the characteristics of Gr−FETs to prepare high-performance biosensors. Haslam’s team in the UK used monoclonal antibodies (Anti-hCG) as the bio-recognition element to prepare a graphene field-effect transistor biosensor for detecting the trophoblastic tumor risk marker human chorionic gonadotropin (hCG), and selected a 0.01 × (100 μM) PBS dilution solution to increase the Debye length to overcome the Debye screening effect, with a detection limit of 1 pg/mL [19]. Zhao Jianlong’s team used monoclonal antibodies as the bio-recognition element to prepare a graphene field-effect transistor biosensor for detecting the colon cancer biomarker carcinoembryonic antigen (CEA), with a detection limit of 100 pg/mL [20]. The researchers selected a 0.001 × (10 μM) PBS dilution solution to increase the Debye length to overcome the Debye screening effect, and the affinity between the monoclonal antibodies and tumor biomarkers remained stable at this concentration. Then, the team further improved the preparation process and pointed out that a dilution solution with an insufficient concentration would affect the detection ability of the sensor; therefore, they selected a 0.01 × (100 μM) PBS dilution solution to increase the Debye length to overcome the Debye screening effect, with a detection limit of 337.58 fg/mL [21].

These studies are highly instructive for detecting PA. Inspired by such detection methods, this study designed and prepared a Gr−FET biosensor combined with monoclonal antibodies for detecting PA.

## 2. Materials and Methods

### 2.1. Experimental Materials and Equipment

#### 2.1.1. Experimental Materials

The following materials were used: anthrax protective antigen (PA), from List Labs in California, USA, stored at 4 °C; anti-protective antigen (Anti-PA) monoclonal antibody, from List Labs in California, USA, stored at 4 °C; Donkey Anti-Goat IgG H&L/HRP (D-HRP) conjugate, labeled with horseradish peroxidase, from Beijing Biosynthesis Biotechnology Co. Ltd., Beijing, China, stored at 4 °C; Yersinia pestis F1 capsular protein, from Beijing Biosynthesis Biotechnology Co. Ltd., Beijing, China, stored at 4 °C; 1 × PBST buffer containing Tween-20, from Beijing Solarbio Science & Technology Co. Ltd., Beijing, China, stored at 4 °C; bovine serum albumin (BSA), from Beijing Solarbio Science & Technology Co. Ltd., Beijing, China, stored at 4 °C; TMB substrate solution, from Beijing Solarbio Science & Technology Co. Ltd., Beijing, China, stored at room temperature; ELISA stop solution, from Beijing Solarbio Science & Technology Co. Ltd., Beijing, China, stored at room temperature; 0.05 M carbonate-bicarbonate buffer (0.05M CB buffer), from Beijing Solarbio Science & Technology Co. Ltd., Beijing, China, pH ≈ 9.6; 1-pyrenebutyric acid N-hydroxy succinimide ester (PBASE), from Beijing Biolab Technology Co. Ltd., Beijing, China, CAS ID: 114932-60-4; Tween-20 reagent, from Beijing Biolab Technology Co. Ltd., Beijing, China, CAS ID: 9005-64-5; 1 × PBS buffer (0.01 M phosphate-buffered saline), from Beijing Solarbio Science & Technology Co. Ltd., Beijing, China, pH ≈ 7.4; ethanol, from Beijing InnoChem Science & Technology Co. Ltd., Beijing, China, CAS ID: 64-17-5; formic acid, from Beijing InnoChem Science & Technology Co. Ltd., Beijing, China, CAS ID: 64-18-6; deionized water (DI), self-made in the laboratory.

#### 2.1.2. Experimental Instruments

The following instruments were used: SpectraMax iD3 microplate reader, manufactured by MD (Molecular Devices, San Jose, USA); Costar microplate, manufactured by Corning (New York, NY, USA); Keithley 2602B digital source meter, manufactured by Keithley (Beaverton, OR, USA) in the United States; BX53M metallurgical microscope, manufactured by Olympus (Tokyo, Japan) in Japan; MPP i5000 multi-function wire bonder, manufactured by K&S(Haifa, Israel).

### 2.2. Experimental Method

#### 2.2.1. Preparation of Detection Device

After transferring the CVD-prepared graphene onto a silicon dioxide/silicon substrate, we proceeded to the placement of the source, drain, and gate electrodes on the graphene using photolithography as part of the preparation of the detection device. To eliminate the likelihood of any interference during the measurement, the surface outside the sensing area was passivated and protected with an SU-8 photoresist. The Gr−FET electrodes were connected to the corresponding electrodes on the integrated circuit board using a wire bonder. Upon installation of the sample chamber, a Ag/AgCl electrode was utilized as the reference electrode.

#### 2.2.2. Detection of Affinity Changes

ELISA experiments were conducted to determine the impact of varying concentrations of PBS solution (1×, 0.1×, 0.01×, 0.001×) on the affinity of Anti-PA and PA. Each concentration was subjected to three independent ELISA tests. PA, with a concentration of 1 μg/mL, was coated onto a 96-well plate using 0.05 M CB, 100 μL/well, followed by overnight incubation at 4 °C. The plate was then subjected to four washes with 0.01 M PBST and blocked with 1% BSA in PBS solution, 100 μL/well, at 37 °C for 1 h. After washing twice with 0.01 M PBST, Anti-PA was diluted to 10 μg/mL, 5 μg/mL, 2.5 μg/mL, 1.25 μg/mL, 0.625 μg/mL, 0.3125 μg/mL, and 0.15625 μg/mL; added to the corresponding wells, 100 μL/well; and incubated at 37 °C for 30 min. After washing four times with 0.01 M PBST, D-HRP was diluted to 1:2000 in PBS solution, added to the wells, 100 μL/well, and incubated at 37 °C for 30 min. The plate was then washed four times with 0.01 M PBST, and TMB color development solution was added. After a 10 min reaction, the reaction was halted with a stop solution. The absorbance was measured using an enzyme-linked immunosorbent assay (ELISA) reader at a wavelength of 450 nm.

#### 2.2.3. Functionalized Sensing Materials

The process of functionalizing sensing materials involved four main steps. Firstly, the Gr−FET surface was meticulously washed thrice with 0.01 × PBS solution and subjected to electrical characterization to obtain the Gr−FET transfer characteristic curve, which was used to determine whether the device met the experimental requirements. Secondly, 20 μL of PBASE, which was dissolved in ethanol to a final concentration of 2 mM, was carefully drop-coated onto the chip surface as a crosslinking agent. The chip was then incubated at room temperature in the dark for 2 h, followed by washing the chip thrice with 0.01 × PBS solution. Thirdly, 20 μL of Anti-PA dissolved in 0.01 × PBS solution to a concentration of 1 mg/mL was drop-coated onto the chip surface as a biological recognition element. The chip was then incubated at room temperature in the dark for 12 h, followed by washing the chip thrice with 0.01 × PBS solution to complete the functionalization of the Gr−FET surface. Finally, 20 μL of Tween-20, which was dissolved in deionized water to a final concentration of 0.05% wt, was meticulously drop-coated onto the chip surface as a blocking agent. The chip was then incubated at room temperature in the dark for 0.5 h, followed by washing the chip thrice with 0.01 × PBS solution to complete the functionalization of the Gr−FET surface.

#### 2.2.4. Signal Acquisition of Biosensor

The process of acquiring signals from the biosensor involved the utilization of the Keithley 2602B digital source meter(Keithley, Beaverton, USA) in conjunction with an integrated circuit card. This combination was used to conduct electrical performance testing of the Gr−FET biosensor. The Keithley 2602B digital source meter, with its user-friendly interface and ease of operation, was deemed fit for the testing requirements. The electrode of the Gr−FET sensor was connected to the integrated circuit card using wire bonding, which greatly facilitated the testing process. The experimental setup involved measuring the gate voltage range from −0.4 V to 1.2 V, with a step size of 0.001 V. The change in I_DS_–V_GS_ (current between the source and drain and the gate voltage) was recorded, while V_DS_ (voltage between the source and drain) was set to 0.5 V.

## 3. Results and Discussion

### 3.1. Physical and Optical Microscopic Characterization of the Detection Device and Sensing Area

**Figure 1:**
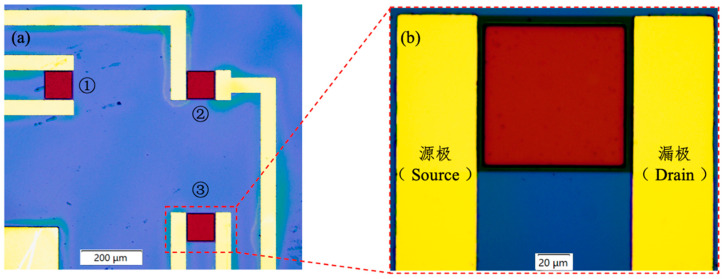
Optical micrographs of Gr−FET sensors combined with monoclonal antibodies. (**a**) A single chip containing three Gr−FET sensors (5× magnification). (**b**) A single Gr−FET sensor (50× magnification).

The dimensions of the chip were 0.5 × 0.5 cm^2^ (length × width, L × W), and each chip was equipped with three Gr−FET sensors. Figure 1 exhibits the optical microscopic depiction of the Gr−FET sensor, synergistically amalgamated with monoclonal antibodies. The sensing area, which is the dark-red square region in Figure 1, was measured to be 100 × 100 μm^2^ (L × W). Following the washing procedure with 0.01 × PBS solution, the graphene in the sensing area remained intact.

### 3.2. Determination of the Optimal Dilution Concentration

**Figure 2:**
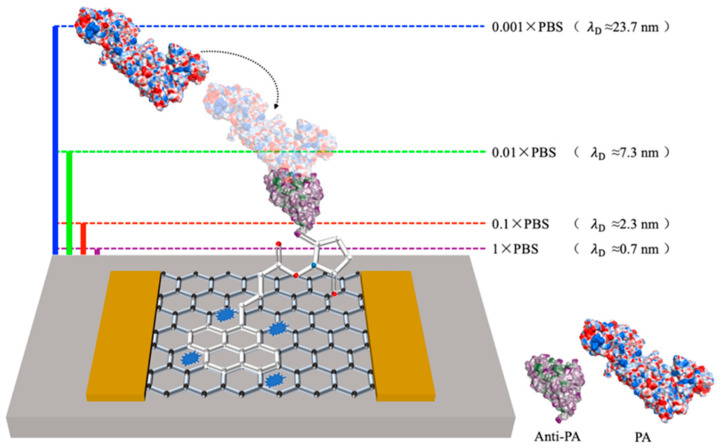
Schematic diagram of Debye lengths corresponding to different concentrations of PBS.

When the test solution comes into contact with the sensing area of the graphene field-effect transistor (Gr−FET) biosensor, charge transfer occurs between the two, forming an electrical double layer (EDL) at the interface with equal and opposite charges [22]. When the distance between anthrax protective antigen in the test solution and the sensing area exceeds the EDL, the charge field effect will be shielded and will not affect the carrier inside the Gr−FET biosensor [23]. This charge-shielding effect is the Debye screening effect, and the distance of charge shielding is the Debye length [24], which can be calculated using Formula (1):

(1) $$\lambda_{D}=\sqrt{\frac{ℇk_{B}T}{q^{2}c}},$$

where ε denotes the dielectric constant of the electrolyte, *k_B_* is the Boltzmann constant, *T* represents the absolute temperature, *q* denotes the charge carried by an electron, and *c* is the ion concentration of the solution. The Debye length is inversely proportional to the ion concentration of the solution, as per Formula (1). For instance, the Debye lengths corresponding to different concentrations of PBS solution are approximately 0.7 nm, 2.3 nm, 7.3 nm, and 23.7 nm [25,26], respectively. In a similar physiological environment, conventional IgG antibodies are much larger than the Debye length at a 1 × PBS concentration, and hence the target substance cannot be detected [27]. The Debye lengths corresponding to different concentrations of PBS solution are shown in Figure 2.

Although the Debye shielding effect can be overcome by diluting the solution to reduce the ion concentration, the diluted solution may cause changes in the structure of Anti-PA and PA, leading to decreased activity and loss of affinity. Additionally, if the concentration of the dilution solution is too low, the conductivity will be affected, and the detection ability of the Gr−FET will be reduced. Therefore, it is crucial to select an appropriate dilution concentration that can overcome the Debye shielding effect, maintain the activity and affinity of Anti-PA and PA to the maximum extent, and maintain good conductivity.

**Figure 3:**
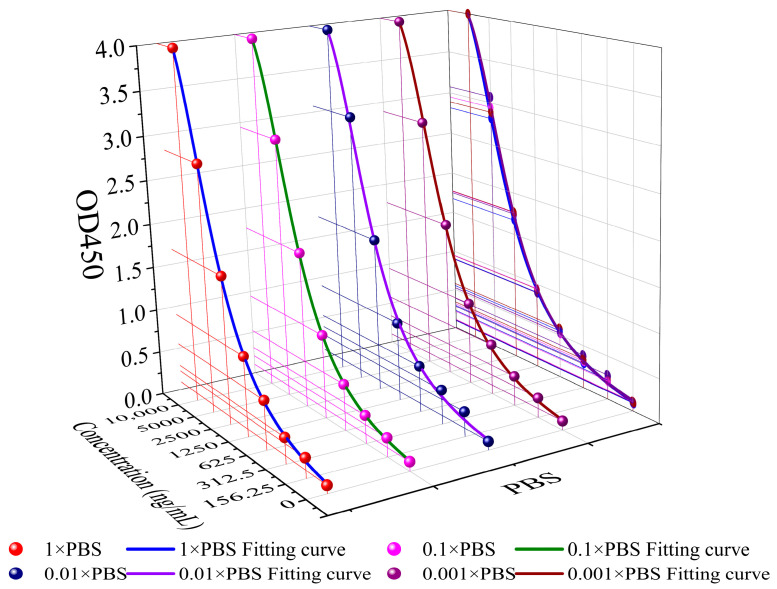
Comparison of the affinity of Anti-PA and PA with different concentrations of PBS solution.

To test the affinity of Anti-PA and PA under different concentrations of PBS solution, three independent ELISA experiments were conducted. The affinity between Anti-PA and PA in the four concentrations of PBS solution is shown in Figure 3. The results indicated that Anti-PA and PA maintained their activity, and the affinity between the two was hardly affected under different concentrations of PBS solution. To ensure a consistent ion concentration in the dilution solution, a concentration of 0.01× PBS solution was chosen as the dilution solution in this experiment, which can overcome the Debye screening effect and ensure that Anti-PA has good binding ability to PA at this concentration.

### 3.3. Changes in Electrical Characteristics during Functionalization Process

**Figure 4:**
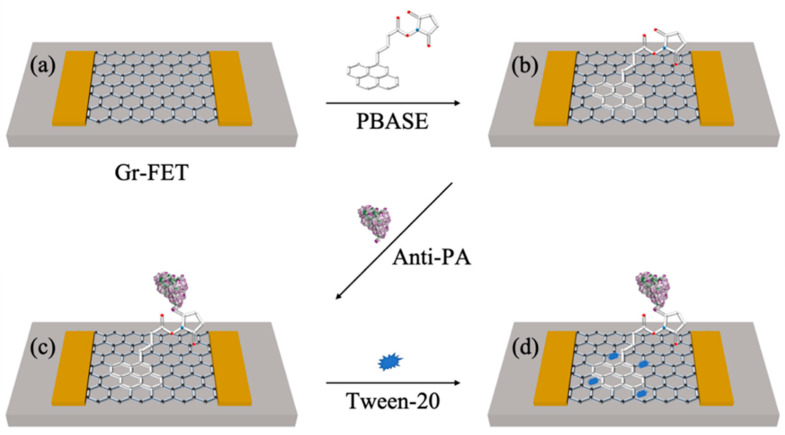
Schematic illustration of the functionalization process of the Gr−FET with Anti-PA. (**a**) Cleaning process of the Gr−FET device. (**b**) PBASE fixed onto the graphene surface as a crosslinker. (**c**) Anti-PA covalently bound to the crosslinker as a biosensing element. (**d**) Tween-20 used as a blocking agent to block excess active sites.

The alterations in electrical characteristics during the process of functionalization, specifically in the Gr−FET sensing area, are due to the absence of active groups on the surface of graphene. This lack of activity makes it challenging to directly immobilize Anti-PA. As a result, PBASE was utilized as a crosslinker to stabilize Anti-PA on the graphene surface of the Gr−FET sensing area. In addition, the Tween-20 reagent was used to block any excess active sites, excluding interference from other non-specific proteins, in order to complete the functionalization of the sensing area. The interaction force between the PBASE pyrene group and the six-carbon ring structure of graphene was used to effectively immobilize Anti-PA onto the Gr−FET sensing area. This interaction force is much stronger than the simple physical adsorption of π–π stacking interaction [28]. The other end of PBASE forms a covalent bond–peptide bond with the amino acid on Anti-PA. A schematic of the functionalization process of the Gr−FET with Anti-PA is shown in Figure 4.

**Figure 5:**
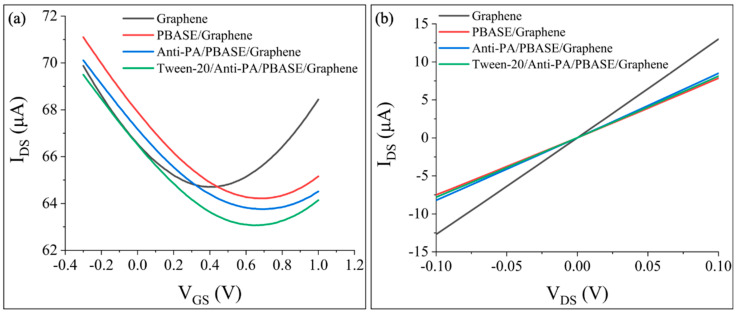
Electrical signal changes in the Gr−FET device during functionalization of monoclonal antibodies. (**a**) Changes in the transfer characteristic curve of the Gr−FET device during the functionalization process. (**b**) Changes in I_DS_–V_DS_ of the Gr−FET device during the functionalization process.

The Gr-FET device was washed with 0.01 × PBS solution and underwent the functionalization process through the PBASE incubation, Anti-PA incubation, and Tween-20 blocking steps. The transfer characteristic curves of the Gr-FET device were collected after each step of the functionalization process. The electrical signal changes in the Gr-FET device during the monoclonal antibody functionalization process are shown in Figure 5.

From Figure 5a, it can be observed that after PBASE incubation, the transfer characteristic curve of the Gr−FET shifted to the right, and V_Dirac_ increased from 420 mV to 710 mV. This indicates that PBASE formed a π–π stacking interaction with the graphene in the sensing area of the Gr−FET and absorbed electrons in the graphene, forming P-type doping, which is consistent with previous reports [17]. After the subsequent incubation with Anti-PA, the transfer characteristic curve continued to shift to the right, and V_Dirac_ increased from 710 mV to 730 mV. This may be due to the isoelectric point (pI) of Anti-PA being greater than 7.4, carrying a positive charge in the 0.01 × PBS solution. When positively charged Anti-PA was fixed in the sensing area, the hole carrier concentration of the field-effect transistor increased, continuing to form P-type doping. Finally, after Tween-20 closure, the transfer characteristic curve shifted to the left, and V_Dirac_ decreased from 730 mV to 680 mV. This decrease in electron absorption in the graphene in the sensing area may be due to the excess active sites being blocked by the reagent. From Figure 5b, it can be seen that during the functionalization process of the graphene in the sensing area, after the antibody was fixed on the graphene in the sensing area using PBASE, the slope (dI/dV) decreased. This slope difference also indicates that the antibody was successfully fixed [29].

### 3.4. Determining the Detection Limit

**Figure 6:**
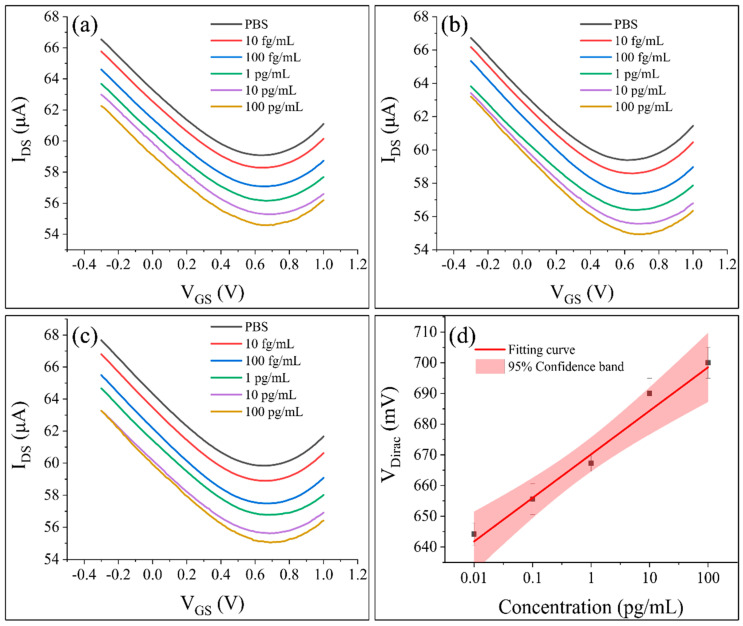
Detection of different concentrations of PA using the Gr−FET biosensor combined with monoclonal antibodies. (**a**–**c**) The transfer characteristics of the Gr−FET biosensor during the 1st, 2nd, and 3rd detections of different PA concentrations, respectively. (**d**) Linear fitting relationship between the PA concentration and V_Dirac_, with a 95% confidence interval.

To ascertain the detection limit, PBS solutions containing PA concentrations of 10 fg/mL, 100 fg/mL, 1 pg/mL, 10 pg/mL, and 100 pg/mL were introduced into the sample pool and incubated for a duration of one minute. The Gr−FET biosensor’s transfer characteristic curves were collated. As the PA concentration increased from 10 fg/mL to 100 pg/mL, the Dirac point gradually shifted to the right. The degree of right shift in the Dirac point increased as the PA concentration increased. The Gr−FET biosensor detected different concentrations of PA, as evidenced in Figure 6. Figure 6a–c illustrate the transfer characteristic curves of the same Gr−FET biosensor detecting different concentrations of PA in the first, second, and third tests, respectively. Figure 6d depicts the linear fitting relationship between the PA concentration and V_Dirac_.

As shown in Figure 6a–c, the Dirac voltage (V_Dirac_) shifted to the right as the concentration of PA increased. The trend of V_Dirac_ shifting to the right increased as the concentration of PA increased. This phenomenon may be attributed to the increase in PA captured by the functionalized graphene surface in the Gr−FET biosensing region. When more PA proteins are captured, the charge carried by PA has a greater impact on the internal carrier of the Gr−FET, and the change in charge density can affect the gate potential of the FET, leading to changes in the FET current output. Additionally, the interaction between PA and Anti-PA can cause a redistribution of the surface charge density, which can further affect the Dirac voltage [30]. Another possibility is that after Anti-PA specifically binds to captured PA, it alters the resistance of the entire Gr−FET biosensing region, leading to a change in the Dirac point. As shown in Figure 6d, when detecting PA with concentrations ranging from 10 fg/mL to 100 pg/mL, V_Dirac_ was linearly correlated with the PA concentration (R^2^ = 0.973), and the actual detection limit was 10 fg/mL.

### 3.5. Verification of Specificity

**Figure 7:**
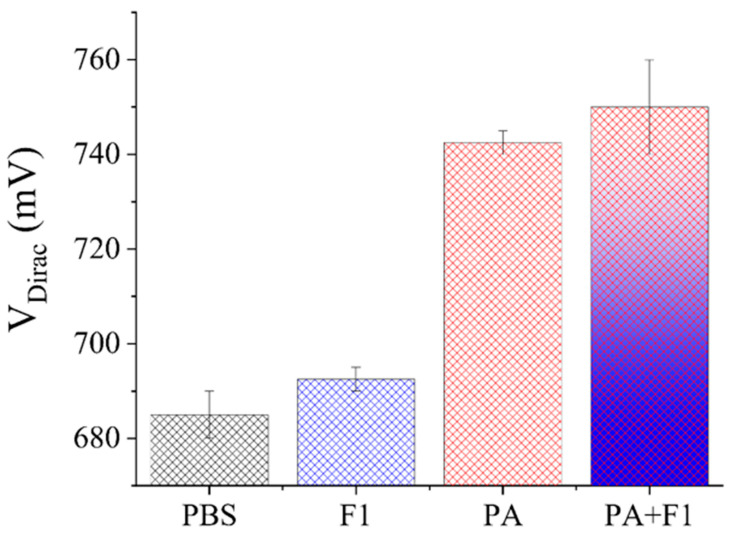
Effect of formic acid dissociation on the repeated detection of PA using Gr−FET biosensors with binding monoclonal antibodies.

To verify the specificity, Gr−FET biosensors were produced to detect 0.01 × PBS solution, 10 pg/mL of Yersinia pestis F1 capsule protein (F1) in 0.01 × PBS solution, 10 pg/mL of PA in 0.01 × PBS solution, and a mixture of 10 pg/mL of PA and F1 in 0.01 × PBS solution. The Gr−FET biosensors, in conjunction with monoclonal antibodies, detected the electrical signal changes in different biomarkers, as depicted in Figure 7.

Figure 7 reveals that there was minimal variation in V_Dirac_ between the Gr−FET biosensor functionalized with Anti-PA when detecting 0.01 × PBS solution and 10 pg/mL F1 solution. When detecting solutions of PA and F1, both at a concentration of 10 pg/mL, the V_Dirac_ change in the PA solution was significantly higher than that in the F1 solution. This phenomenon may be attributed to the fact that the Anti-PA functionalized on the graphene surface of the Gr−FET biosensor only binds to PA and not to F1. When detecting a mixed solution of PA and F1 at a concentration of 10 pg/mL, V_Dirac_ was slightly higher than that in the solution containing only 10 pg/mL of PA. At the same time, it was observed that the Gr−FET biosensor also responded to the F1 solution at a concentration of 10 pg/mL, which may have been due to a small amount of F1 depositing on the Anti-PA surface of the sensing area when the solution flowed through, thereby affecting the internal carrier of the Gr−FET. The different electrical changes produced by the sensor for different target molecules at the same concentration indicate that this biosensor has a certain specificity for detecting PA.

### 3.6. Verification of Reproducibility

**Figure 8:**
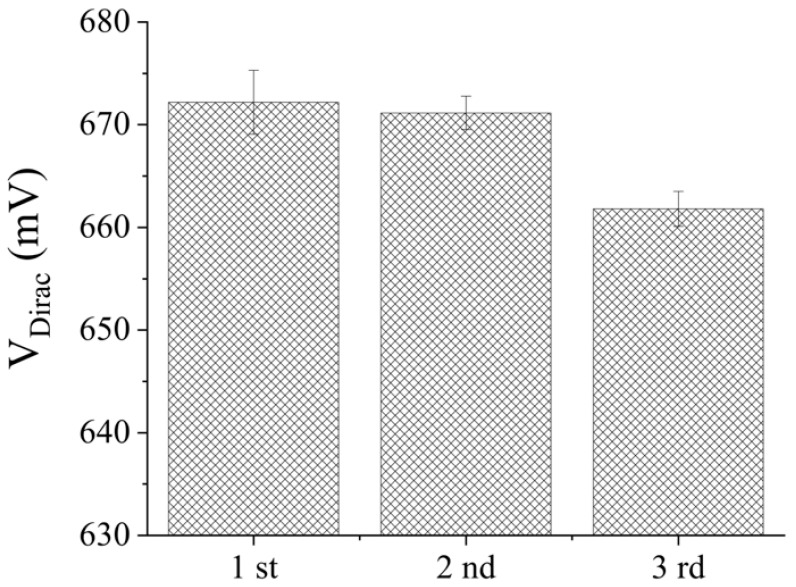
Electrical signal changes detected by the Gr−FET biosensor combined with monoclonal antibodies for different biomarkers.

In order to evaluate the reproducibility of the Gr−FET biosensor, 1 M formic acid solution was employed to dissociate the specific binding between Anti-PA and PA. Following the initial PA detection, the remaining liquid was removed from the sample pool, the sample was washed thrice with 0.01 × PBS solution, 10 μL of formic acid solution was added, and then the sample was left to stand for 1 min. The sample was then quickly washed thrice with 0.01 × PBS solution, and a concentration of 1 pg/mL PA in 0.01 × PBS solution was detected. This process was repeated three times to observe the V_Dirac_ offset by transferring the specific curve. The effect of formic acid dissociation on the repeated detection of PA by the Gr−FET biosensor is illustrated in Figure 8.

As shown in Figure 8, the signal attenuation in the second measurement was less than 1% compared to the first measurement after methanoic acid dissociation, and the signal attenuation in the third measurement was less than 5%. This indicates that the biosensor has the potential for repeated use in detecting PA.

## 4. Conclusions

In summary, our study successfully developed a biosensor based on a Gr−FET device constructed on CVD-grown graphene for the detection of ultra-low concentrations of PA, an important biomarker of anthrax. The biosensor demonstrated high sensitivity and specificity, with a detection limit as low as 10 fg/mL, surpassing that of the ELISA detection method. Our analysis also revealed that the monoclonal antibody’s affinity to PA was not affected by the PBS dilution concentration. Furthermore, the biosensor demonstrated good specificity in distinguishing between F1 and PA samples with significantly different electrical signals. The signal attenuation after three repeats of formic acid dissociation was less than 5%, indicating good repeatability. Our biosensor presents a novel and effective approach for the detection of PA, which has great potential for applications in clinical diagnosis, food safety, and environmental monitoring. These findings contribute to the development of biosensors for the detection of ultra-low concentrations of biomarkers, which are crucial for early diagnosis and disease monitoring.

## Data Availability

Not applicable.
